# Understanding modern contraception uptake in one Ethiopian community: a case study

**DOI:** 10.1186/s12978-018-0550-3

**Published:** 2018-06-20

**Authors:** Erica Sedlander, Jeffrey B. Bingenheimer, Mark C. Edberg, Rajiv N. Rimal, Hina Shaikh, Wolfgang Munar

**Affiliations:** 1 Department of Prevention and Community Health, The George Washington University, Milken Institute School of Public Health, 950 New Hampshire, Washington, DC USA; 2 Department of Global Health, The George Washington University, Milken Institute School of Public Health, 950 New Hampshire, Washington, DC USA

**Keywords:** Family planning, Contraceptives, Health extension worker, Ethiopia, Sub Saharan Africa, Community based interventions

## Abstract

**Background:**

In the last decade, the proportion of Ethiopian women using contraceptive methods has increased substantially (from 14% in 2005 to 35% in 2016 among married women). Numerous factors have contributed to the increased uptake. An important one is the implementation of the Health Extension Program, a government-led health service delivery strategy that has deployed more than 38,000 health extension workers (HEWs) throughout the country. Key mechanisms underlying the success of this program are not well understood. Using a case study approach, the goal of this study is to describe how key features of local contexts, community perceptions, and messaging by HEWs have contributed to the increased use of modern contraception in one community in Ethiopia.

**Methods:**

We conducted focus groups and individual interviews with men, women, adolescents, and key informants, including (HEWs), in Oromia, Ethiopia. We used a random sampling protocol to recruit all participants except key informants, with whom purposive sampling was used to ensure participants were knowledgeable on family planning in the village. Interviews were audio recorded, translated, transcribed, and then analyzed using applied thematic analysis and NVivo v.11 qualitative research software.

**Results:**

We identified four themes that may explain uptake of contraception: (1) HEWs are seen as trusted and valued community members who raised awareness about family planning; (2) the HEW messaging that contraception is useful to space pregnancies among married women was effective; (3) the message that spacing is healthy for mother and child was also effective; and (4) communicating to the entire community (including men, women, adolescents, and religious leaders), contributed to changing attitudes around contraception.

**Conclusion:**

The four aspects of the Health Extension Program approach increased uptake of contraception in our sample. In contexts where community health workers are valued by the health systems and local communities they serve, this type of approach to widening modern contraception use could help increase uptake and address unmet need. Understanding these granular aspects of the program in one local context may help explain how use of contraception increased in the country as a whole.

## Plain English summary

In the last decade, the proportion of Ethiopian women using contraceptive methods has increased substantially (from 14% in 2005 to 35% in 2016 among married women). One of the reasons for this increase is the government-initiated health program that hired and trained over 38,000 female Health Extension Workers (HEWs). Although this program has shown a high level of success in increasing the use of family planning methods, little is known about how the program works on the ground. In this study, we wanted to understand how uptake of contraceptive methods occurred in one community in Ethiopia. To do so, we conducted interviews and focus groups in the Oromia region of Ethiopia. We found four main themes: (1) HEWs are seen as trusted and valued community members who raised awareness about family planning; (2) the message that contraception is useful to space pregnancies was effective; (3) the message that spacing is healthy for mother and child was also effective; and (4) communicating to the entire community (including men, women, adolescents, and religious leaders), contributed to changing attitudes around contraception. Understanding these specific components of the program in one local context may help explain how use of contraception increased in the country as a whole.

## Background

Between 2005 and 2016, total fertility in Ethiopia dropped and contraceptive use increased significantly. According to Demographic Health Surveys (DHS), total fertility dropped from 5.4 in 2005 to 4.1 in 2014. Additionally, married women more than doubled their use of modern contraception from 14% in 2005 to 35% in 2016 [1, 2]. Knowledge of modern contraceptive methods among women is almost universal: It increased from 80.0% in 2005 to 97.1% by 2011 [3, 4]. Various factors have been proposed to explain this increase in contraceptive use, including growing political will, substantial external funding, non-governmental and public-private partnerships, and implementation of the Health Extension Program (HEP) to train health extension workers (HEWs) to work at community health posts [5].

Ethiopia’s large, government-sponsored HEP, operationalized in 2004, brings health education and a range of primary care health services to rural areas. Some of the services include personal hygiene, water sanitation, disease prevention and control, maternal and child health, and family planning [5]. Across Ethiopia, some 38,000 HEWs work in over 1500 villages and serve as an intermediary between the community and the government-provided primary health services to expand health infrastructure and services [6]. In contrast to the prevailing practice in other low-income countries, HEWs in Ethiopia are salaried and receive one full year of intensive training based on a curriculum developed by a team of national and international experts [7, 8]. The recruitment criteria require that candidates be females who have at least a secondary school education; are 18 years or older and residents of the *kebele*, or village, where they will work (a community made up of about 500 households); and are committed to returning to their respective *kebele* after training is complete. The HEWs are recruited from within their own community to ensure delivery of locally needed care and to facilitate trust between the community and the HEWs [9].

The program adopts a diffusion model, which posits that community change occurs incrementally, by training early adopters first and then moving to the next group that may be ready to change [10]. One example of this is the Health Development Army (HDA), a component of the HEP introduced in 2012. Within the HDA, HEWs train “model families” -- households that adopt specific healthy behaviors and receive 96 h of training. Model families are considered early adopters of desirable health practices and become leaders of a group of five families known as the “one to five network,” who subsequently form a group of 25 to 30 households within a village [11].

Several studies have identified key elements for the effectiveness of the HEP platform, including the use of data to monitor performance, clearly defined roles among various program actors and stakeholders, and standardized support from the Minister of Health for HEWs [6, 11]. Specifically, the training and deployment of HEWs have been shown to reduce barriers, increase access, and help change fertility and family planning social norms in Ethiopia [12–14].

Although the success of the HEP has been lauded by many international organizations, to our knowledge, no study has examined the specific components within the family planning program administered by the HEWs that led to select achievements [15–17]. Several calls have been made to document the process of the Ethiopian HEWs as a successful service delivery model and to reduce the implementation gap for other health facilities and communities [5, 8]. While our initial focus of this study was more broadly focused on how uptake of contraception increased in one community, our data quickly revealed the substantial influence that the HEWs had on uptake. Therefore, our findings are primarily focused on their influence and specific aspects of the HEP program.

In this paper, adopting a case study approach, we describe *how* Ethiopia’s public sector HEWs helped to increase modern contraception in one rural district. We focus on the following questions:

1. Which behavior change outcomes were targeted by the HEWs?
2. Which framing and key messages were communicated to the community?
3. What approach was used by HEWs to engage the local community?

## Methods

In July and August 2016, we conducted a case study of one community in Ethiopia. The goal of case study research is to understand the complexity of the behavior patterns of the catchment area or bounded system (in this case, the geographic community) [18]. To understand how modern contraceptive use increased in this community, we conducted five focus groups and 13 individual interviews with men, women, adolescents and key informants in one rural area in Oromia, Ethiopia (*n* = 59). (See Appendix #1 for number of total interviews and focus groups for each type of participant). Key informants included HEWs, teachers, and religious leaders. All interviews were conducted face-to-face in the local languages, Afan Oromo or Amharic, by native speakers trained in qualitative interviewing, using pretested and open-ended discussion guides that covered where and how women receive information about family planning and attitudes around use of family planning. To explore family planning norms in a less personal and threatening way within the focus groups, we used vignettes, short stories about hypothetical characters who live in a rural village in Oromia [19]. We chose qualitative methods to better understand the local approaches used to increase modern contraceptive uptake in one community and to understand how these approaches were carried out on the ground. We chose to two types of individual interviews to gather different information: key informant interviews focused on a broader profile of village-level patterns; and life-history interviews obtained in-depth narratives about experiences with childbearing, contraceptive use, and decision-making factors.

### Setting

The Oromia region (population 279,639) is one of the nine ethnically based regional states of Ethiopia. Over 90% of its residents live in rural areas [20]. We chose Oromia because it exemplifies the dramatic increase in family planning use and drop in fertility. In 2005, the total fertility rate in Oromia was 6.2, and 12.9% of currently married women ages 15–49 reported use of family planning [4]. In 2014, the percentage of married women using family planning more than tripled, with 39.4% reporting use of family planning, and the total fertility rate dropped to 4.4, higher than the national average of 4.1 [2]. In Oromia, we collected data in one verdant, hilly and rural *gare* (a community made up of approximately 90 households). The gare is located in the Yebu District of Jimma Zone, Southwestern Ethiopia which is approximately 200 miles southwest of Addis Ababa. We chose a rural gare because it is more representative of the predominantly rural Oromia region. Focus groups were conducted in the village primary school and interviews were conducted outside of homes or at the HEWs stations/health posts.

We used a random sampling procedure to select participants for focus group discussions and life history interviews. Research team members conducted a household enumeration activity in the *gare* (an area of approximately 90 households). Based on the number of individuals needed for focus group discussions and interviews, against a sampling frame that consisted of the entire gare, we used a proportional skip pattern that began with a randomly selected initial participant in order to identify households from which to select every succeeding participant for each category (e.g., mothers, adolescent girls). We used purposive, critical case sampling to select key informants based on their level of knowledge about family planning in the village. [21] For the purpose of this study, we define community as the *gare* from which we recruited participants.

### Analysis

Interviews and focus group discussions were audio-recorded, transcribed, and translated to English by our research partners in Jimma Ethiopia. Transcripts were uploaded to NVivo v.11 qualitative software for analysis [22]. We analyzed transcripts using applied thematic analysis, an inductive set of procedures designed to identify and examine emerging themes from conceptual data [23]. Following the procedures outlined by Bradley, Curry and Devers (2007), we used both inductive and deductive coding [24]. Specific a priori codes were used to identify text related to attitudes and practices around family planning, and additional codes were then generated based on new themes that emerged. Two experienced qualitative researchers independently reviewed transcripts to develop an initial codebook and modified the codebook as themes emerged. One researcher coded all transcripts and another coded 20% to ensure consistency across coding. They met over the course of the analysis to discuss codes and reconcile discrepancies. Using NVivo v.11, we identified themes by comparing codes and content across sources, and by running specific word queries, associations between themes, and creating hierarchal visual displays of codes to identify linkages and patterns in the data. This study was approved by the Institutional Review Boards of Jimma University in Ethiopia and The George Washington University in the United States.

## Results

Participants ranged in age from 14 to 55 years, the median and mean ages being 18 and 23, respectively. Most participants (68.5%) were female. All identified themselves as Muslims and as members of the Oromo ethnic group. We identified four main themes that may help explain uptake of modern contraception in Oromia: (1) high acceptance of and impact on uptake of family planning services provided by HEWs in the community; (2) a focus on changing behaviors to increase spacing between births; (3) framing the spacing message as “healthy” for mother and baby but only targeting married women who have already given birth; and (4) delivering the message to everyone in the community, including men, women, adolescents, and religious leaders. Themes are further described below with quotes.

### Theme 1: HEWs are seen as trusted and valued members of the community responsible for increasing use of family planning

We found that most participants valued the increased awareness and use of family planning methods, which they primarily attributed to the HEWs. Many participants mentioned that this was a fairly new and positive change within their community. According to one adolescent boys’ focus group participant, “*Since the health extension workers have been teaching and giving advice throughout the meetings, mothers started to use contraceptives. Before five/ten years, people didn’t have awareness about using these contraceptives*.”

The HEWs were also perceived as trusted community members. In the men’s focus group, one participant said, “*Previously people consulted traditional midwifery which resulted in so many problems but now they [women] don’t discuss the issue with their family or relatives. Rather they talk with the health workers. So they even discuss the secrets that they don’t tell their husbands with the health extension workers*.”

### Theme 2: HEWs focused on the use of contraception to “space births” among married women

Our data illustrate three main sub-themes within family planning behavior change. We describe these themes on a spectrum from least acceptable (substantial resistance) to the most acceptable (a relative openness) within the community. The themes are the following: (1) substantial resistance to the idea of delaying the initiation of childbearing after marriage; (2) some resistance to the idea of deliberately stopping childbearing or limiting; and (3) a relative openness to spacing births.

### Substantial resistance to the idea of delaying the initiation of childbearing after marriage

Our data show that all newly married couples in our sample experience a great deal of internal and normative pressure to conceive soon after marriage. A mother in a focus group stated, “*After she gets married having children is necessary; so that, she does have the first child after getting married, and then she can stay some years to have the second child. But, she can’t wait initially without children*.” A boy from a focus group supported this cultural norm, expressing, “*If she has planned to get marriage, what will follow is to have a child*.”

### Some resistance to the idea of deliberately stopping childbearing or limiting

Clearly, delaying birth after marriage does not align with the community norms. A mother in a focus group reported that there is more openness to limiting total number of children, “*The mistake we did with lack of awareness at previous times is not available now and everyone knows to limit the number of children they have*.” However, according to a midwife, some religious push back still lingers in regards to limiting, “*In many households about this regulating the number of children is not common because of considering child as a gift from Allah….they don’t want to limit*.”

### A relative openness to spacing births

Conversely, once the first child is born, couples are more flexible in making decisions about family size and spacing. A mother’s statement in a focus group illustrates the rigidness of giving birth immediately after marriage but the flexibility in regards to spacing, “*After she gets married having children is necessary; so that, she does have the first child after getting married, and then she can stay some years to have the second child.*” In this community, HEWs focused on increasing the practice of spacing after the first birth. All participants knew of and accepted family planning to space childbirth. As a result of HEWs efforts, in this community, spacing is not only perceived as an accepted practice, but it is also a highly regarded one. A mothers’ focus group participant said, “*You know, it was great problem in the past when we didn’t know. But, now we are spacing our births and most women are doing so in our community*.” Clearly, this strategy of targeting married women who have already given birth and primarily promoting one behavior, spacing, is effective within this community.

### Theme 3: Messaging that “spacing is healthy for mother and baby” among married women resonated within the community

In regards to changing behavior to space births, HEWs communicated that spacing is a “healthy” practice for both mother and baby. One adolescent girl’s statement during a focus group illustrates this common sentiment: “*Giving birth frequently without gaps affects the mothers physical health and their health in general. Spacing or limiting is important for children’s development and for mothers as well*.”

Additionally, spacing for “five years” was cited by almost all participants who referenced a specific number of years to space; this may likely reflect the consistent message disseminated by the HEWs. A mother from an individual interview stated that the HEWs provide awareness to the whole community about contraception and the health benefits of spacing, “*Now I am left only with one year; I planned to give birth after five years. If the gap between children is narrow, it hurts both preceding child and the newborn*.”

### Spacing and use of contraception for married women only

Although participants were not asked about use of contraception among adolescents specifically, throughout the interviews, all participants discussed use of contraception as commonplace among married women only. In the adolescent girls’ focus groups, all participants stated that they were aware of contraception and discussed the benefits in reference to married women, not themselves. As one health extension worker said, “*Almost all adolescent boys in this village were educated and they have positive thoughts about the benefits of family planning.”* However, several participants stated that family planning education and services for adolescents were inadequate though a few felt that it was sufficient. One health extension worker reported that the HEP curriculum includes a module on adolescent reproductive health but that it is not emphasized.

“Reproductive health of adolescents is one of the health extension packages and it concerns awareness for the adolescents. But it is not practically dealt as it is put on the packages. There is initiation on some of the places on youth friendly services, but it is still not wide. This gap is created from the higher policy level.”

We observed a discrepancy between awareness about family planning as reported by the adolescents in the focus groups and actual use among them. As one HEW said, “*They get some information at school and from health extension professionals working in the community, but; adolescents visit the health facility when they face problems rather than for consulting what to do to protect themselves*.”

### Theme 4: A whole-community approach to HEWs engagement may have been critical for changing fertility norms and attitudes about the use of modern contraception

According to all participants who mentioned the target population, family planning education offered by the HEWs was made available to all members of the community, including men, women, adolescent boys and girls, and religious leaders. Including men in the dialogue may be particularly important because most participants stated that the husband is the “decision-maker” within a family. A participant in a mother’s focus group said, “*It is husband’s word that is given great place in using contraceptives. Husbands were not positive at initial period, but as time goes on, they see its advantage and they are encouraging us to take it. Now those health professionals are also giving advice to males as well*.” Building upon this, according to one HEW, they also help mediate differences between couples in the use of family planning, “*If, she faced challenge from her husband in using family planning, there will be negotiation with him by the help of health extension professionals*.”

In addition to the influence from the husbands and religious leaders, another HEW said that although some women make decisions without external influence, “the *majority of the families involve the mother-in-law in decisions around some of the issues in a family*,” including use of family planning. Given these multiple influencers, communicating to all members of the community, not solely to women as contraceptive users, may be a critical step for acceptance within the community.

Respondents also highlighted the role that HEWs play in influencing the perceptions of religious leaders about spacing, which is critical given that, according to one health extension worker, religious leaders have “*a lion share role in terms of influencing people to use family planning*.” According to another health extension worker, “*As time goes on, religious leaders were called at health facilities and they were given repeated training and awareness to convince them. After that, they never complained about using contraceptives to space birth at all. Fortunately, now a days religious leaders, husbands, women as well as community as a whole understand the advantage of birth spacing and feel happy about it*.”

## Discussion

Our findings illuminate some key areas where HEWs effectively increased use of family planning within one Ethiopian community. The HEWs have helped make family planning a widely accepted practice to space births among married women who have previously given birth. Participants reported that spacing was “healthy” for mother and baby and that it improved their quality of life, thus reflecting positive valuation of HEWs and, in turn, increasing trust in them as individuals and in the services they provide. The HEWs educate not just women but men, teachers, and religious leaders about family planning. Our data suggest that changing norms related to the social acceptance of discussing family planning with husbands and religious leaders may be a critical step for reducing normative barriers to the adoption of modern contraception. Some participants also mentioned that practices related to family planning for adolescents have not been prioritized. We encountered conflicting accounts about whether or not adolescents receive adequate sexual health education. Adolescents were familiar with contraception, but they spoke about it solely in regards to married women, suggesting that there may be a general resistance to adolescents using it, perhaps because of a presumption that unmarried adolescents should not be sexually active.

Our finding that the HEP may have contributed to increasing the use of contraception is in line with the Health and Health Related Indicator report issued by the Ethiopia Federal Ministry of Health that shows the contraceptive acceptance rate increased significantly following the launch of the HEP, from 23% in 2004 to 61.7% in 2011 [25]. A 2011 evaluation of the HEP found that that family planning practices were rated highest among community members using services administered by HEWs, and the leading reason that they visited HEWs was for family planning [26]. Other studies have found that HEWs are trusted and valued members of the community, and that they are the primary communicators and educators about family planning [9, 12].

A 2015 review of community-based reproductive health interventions for young married couples in developing countries included eight studies from Malawi, India and Nepal. While the studies varied in their targeted behavioral outcomes, target population, and to whom they delivered key messages, the review found that the most successful interventions included counseling for the women themselves, husbands, family members, and the community as a whole [27]. Other studies have shown that including men in discussions around family planning is critical [28, 29]. Similarly, including religious leaders in the dialogue may shift community norms. A study in Rwanda found that religious leaders were named as the primary community members who changed perceptions about family planning [30]. In certain contexts, gaining approval from religious leaders may be key for shifting norms and practices, if such leaders are perceived as trusted and important influencers in the community.

Our findings that adolescent sexual health education and use of family planning need improvement are echoed in the literature. One study suggests that health workers in Ethiopia (primarily consisting of HEWs) may not have the required training to effectively communicate with adolescents [31]. The same study found that health workers have mixed attitudes about providing family planning services to unmarried adolescents; nearly one third had negative attitudes and almost half had unfavorable responses about providing reproductive health services to unmarried adolescents. According to the Family Planning 2020 website, at the 2012 London Family Planning Summit, the Ethiopian Minister of Health pledged to improve “the needs of adolescent girls” and “to expand youth friendly services” [5, 32]. Clearly, there is a need but also a desire to improve services for adolescents.

### Limitations

This work has several limitations. Participants were from one rural area in Ethiopia and are not representative of all Ethiopian perspectives. However, our focus groups included a diverse sample of participants and the majority of sub-Saharan Africa is rural, so our findings may be applicable to similar settings. Additionally, we only included one community. A comparison of different approaches to family planning from different communities using the HEP curriculum may yield helpful information. This is especially critical given that HEWs are selected from their own community and tailor their approach to locally appropriate content. Another limitation is that focus group-based study designs may produce selection biases; those who choose to participate in research may be a different population than those who do not. However, to reduce this threat to validity, we utilized a random sampling approach as described in the methods section.

Social desirability bias is another threat. Because information related to family planning and the HEWs are government services and employees, respectively, we tried to minimize this potential bias by reminding participants that discussions were anonymous, using vignettes within focus group guides for participants to be able to speak about someone hypothetical, and gathering data from a variety of participants and comparing responses. Although we purposefully conducted interviews and focus groups with a wide range of participants, we do not have a large number of data sources from each demographic group. Additionally, although we describe how participants learn about family planning and which messages they hear, we cannot account for all of the sources of information that may be influencing this community, which may include the internet or public health campaigns. Lastly, we did not specifically ask questions about family planning practices among unmarried women or adolescents.

### Future research

While focusing on married women was successful in this community, future research should include adolescent and unmarried women’s perceptions of family planning among themselves, not just married women. While there are adolescent sexual health modules within the initial HEW curriculum, prioritizing these sub-populations more or a continued education module for the HEWs after targeting married women may be a logical approach [23]. Additionally, this study highlights a single Ethiopian community, but a comparison of strategies across select villages would illustrate how other approaches may be similarly effective or ineffective. Furthermore, additional information on the HEP training and curriculum development may highlight a nationwide incremental strategy versus a local approach. Lastly, longitudinal qualitative data could examine how HEWs adapt their approaches (e.g., a change in the sub-population they target, the behavior and messaging) as the attitudes and behaviors around family planning shift within the community where they work.

## Conclusion

To the extent that the HEWs have been effective in changing attitudes and behaviors related to family planning, their success may be attributable in part to a strategy of incremental change. It appears that the HEP focuses on less controversial approaches to increasing family planning uptake (e.g., contraception among married women *after* their first child to space births). Even if the education remains focused around spacing for married women only, the HEWs in this community in Oromia include men and religious leaders in the discussion, who comprise a seemingly less traditional group to bring into conversations about contraception use. Although religious leaders, adolescents, and unmarried women are all included in community dialogues about family planning, unmarried women and female adolescents may not be included as primary contraception users themselves, if social norms do not approve of their sexual activity. Our study shows that the HEP as administered in this community may have worked because of these approaches that appear to be effective. Other studies could use this type of qualitative examination to understand potentially amenable changes in attitudes and behaviors within a community.

Clearly, as shown by impact evaluations and corroborated by this study, HEWs are an effective vehicle to transfer information and ideas. The extent to which HEWs can also be used to push a more controversial public health agenda – to provide and promote access to information, education, and contraception among unmarried women and adolescents – remains to be seen. Changing social norms is a difficult endeavor and understanding where to begin and which subsequent step to take is critical. Focusing efforts on acceptance of family planning for spacing among married women can be an effective initial approach.

## Appendix

### Appendix #1: Interview and Focus Group Count

Total Interviews and Focus Group Participants (*n* = 59).

Key Informant Interviews (HEWs, religious leaders, teachers) (*n* = 8).

Five Focus Groups (adolescent boys, adolescent girls (2), men, women) (*n* = 46).

Life History Interviews (married women with and without children) (n = 5).

## Abbreviations

DHS: Demographic health survey
HDA: Health development army
HEP: Health extension program
HEW: Health extension worker
